# Silencing long non‐coding RNA MEG3 accelerates tibia fraction healing by regulating the Wnt/β‐catenin signalling pathway

**DOI:** 10.1111/jcmm.14229

**Published:** 2019-04-06

**Authors:** Yu‐Bao Liu, Lu‐Pan Lin, Rui Zou, Qing‐Hua Zhao, Fu‐Qing Lin

**Affiliations:** ^1^ Department of Orthopaedics Luhe People’s Hospital of Nanjing Nanjing P.R. China

**Keywords:** fracture healing, long non‐coding RNA, maternally expressed gene 3, Tibia fracture, Wnt/β‐catenin signal pathway

## Abstract

As fracture healing is related to gene expression, fracture healing is prospected to be implicated in long non‐coding RNAs (lncRNAs). This study focuses on the effects of epigenetic silencing of long non‐coding RNA maternally expressed gene 3 (lncRNA MEG3) on fracture healing by regulating the Wnt/β‐catenin signalling pathway. Genes expressed in fracture were screened using bioinformatics and the subcellular location of MEG3 was determined using FISH. Next, we successfully established tibia fracture (TF) models of C57BL/6J and Col2a1‐ICAT mice and the effect of silencing lncRNA MEG3 on fracture healing was detected after TF mice were treated with phosphate buffer saline (PBS), MEG3 siRNA and scramble siRNA. X‐ray imaging, Safranin‐O/fast green and haematoxylin‐eosin (HE) staining and histomorphometrical and biomechanical analysis were adopted to observe and to detect the fracture healing conditions. Additionally, the positive expression of collagen II and osteocalcin was examined using immunohistochemistry. At last, in the in vitro experiment, the relationship of MEG3 and the Wnt/β‐catenin signalling pathway in fraction healing was investigated. MEG3 was located in the cell nucleus. In addition, it was found that MEG3 and the Wnt/β‐catenin signalling pathway were associated with fraction healing. Moreover, silencing MEG3 was proved to elevate callus area and maximum bending load and to furthermore enhance the recanalization of bone marrow cavity. Finally, MEG3 knockdown elevated levels of Col10a1, Runx2, Osterix, Osteocalcin, Wnt10b and β‐catenin/β‐catenin whereas it reduced p‐GSK‐3β/GSK‐3β levels. Taken together, our data supported that epigenetic silencing of lncRNA MEG3 could promote the tibia fracture healing by activating the Wnt/β‐catenin signalling pathway.

## 1 INTRODUCTION

Fractures, considered as the most common traumatic injuries in humans across the world,^1^ are a complicated process in which mechanical forces are necessary for the regeneration of bone tissues.^2^ Tibia fracture (TF) is known as the most common form of fracture injury and its indications are characterized in terms of site, type and local fracture mechanisms.^3^ Inflammation and granulation tissue formation, callus formation and remodelling are the three known major stages of fracture healing.^4^ The rate of non‐union after treatment of TF ranges from 5%‐33%.^5^ It has been reported that when antero‐posterior and lateral radiographs demonstrate a bridging callus and/or the fracture stability, fractures are primarily healed.^6^ A basic phenomenological model differentiates the formation of new bone through the endochondral and intramembranous ossification pathway.^7^ During the complex morphogenetic process, cartilage‐ and bone‐forming cells work in coordination to restore skeletal integrity and their activity is regulated by local growth factors and cytokines.^8^ Fracture repair can restore impaired skeletal organ to its pre‐injury cellular composition, whereas biomechanical function and structure (about 10% of fractures) are not known to heal normally.^9^ Despite modern advances in fracture care, deep (implant‐related) infection remains to be a problem for the treatment of TF.^10^ Fracture healing with a variety of molecular and cellular events is involved in proliferation, differentiation and apoptosis.^11^ Therefore, fracture healing requires the controlled expression of thousands of genes.^12^

In view of the relationship between fracture healing and gene expression, fracture healing is considered to be associated with long non‐coding RNAs (lncRNAs).^13^ LncRNAs, a group of non‐coding RNAs composed of >200 nucleotides,^14^ have recently emerged as a novel group of non‐coding RNAs, capable of regulating gene expression.^15^ Maternally expressed gene 3 (MEG3) is regarded as a maternally expressed imprinted gene, which refers to a large non‐coding RNA in which microRNAs (miRNAs) and small nucleolar RNAs are also hosted.^16^ MEG3 located on chromosome 14q9 has been known to exert great effects on some cancers.^17^ Among the complex and numerous cellular signalling pathways that are activated and are paramount for bone remodeling and development, a vital event may be credited to the Wnt/β‐catenin ligand‐induced intercellular pathway.^18^ In addition, β‐catenin, known as the nuclear accumulation of the pathway, has been previously reported to play vital roles in osteoblast differentiation and osteogenesis.^19^ In addition, the regulation of the Wnt/β‐catenin signalling pathway has been reported to play a function in normal fracture repair.^20^ In terms of the relationship between fracture healing and lncRNAs or the Wnt/β‐catenin signalling pathway, the current study aims to explore the effects of epigenetic silencing of lncRNA MEG3 on fracture healing by regulating the Wnt/β‐catenin signalling pathway.

## 2 MATERIALS AND METHODS

### 2.1 Ethics statement

The current research was approved by the Animal Ethics Committees of Luhe People's Hospital of Nanjing in accordance with the principles of animal protection and the relevant provisions of national animal welfare and ethics. All efforts were made to minimize the suffering of the animals included in the study.

### 2.2 Bioinformatics prediction

Non‐union fracture bone microarray profiles (GSE494) and probe annotation from the Gene Expression Omnibus (GEO) database (http://www.ncbi.nlm.nih.gov/geo) were detected using the [HG_U95B] Affymetrix Human Genome U95B Array. The datasets were processed with background correction and normalization using the Affy package of the R software.^20^ Differentially expressed lncRNAs were screened from the profiles using lineal model‐empirical Bayes statistics combined with *t* test using the Limma package.^21^ The RCricos package of the R software^22^ was adopted in order to determine the location of the first ten differential expressed genes (DEGs) in chromosome and the expression in samples and the LncATLAS website (http://lncatlas.crg.eu/) was employed in order to predict the location of lncRNA. The RAID v2.0 website (http://www.rna-society.org/raid/index.html) was adopted to find the protein binding to the target lncRNA and Blast was used to compare whether there was complementary pairing sequence between the promoter region of the target gene and the lncRNA (https://blast.ncbi.nlm.nih.gov/Blast.cgi).

### 2.3 Dual‐luciferase reporter gene assay

The promoter sequence of Wnt10b and full length of the genes were obtained from the NCBI database (http://www.ncbi.nlm.nih.gov/gene). Subsequently, the full length of the 3’‐untranslated regions (3'UTR) of the wild‐type (WT) Wnt10b was cloned and amplified. The products of polymerase chain reaction (PCR), using the endonuclease sites SpeI and Hind III, were cloned into the downstream polyclonal site of pmirGLO (Promega, WI, USA) Luciferase gene in order to construct the pWnt10b‐Wt group. Through comparison of the binding site between MEG3 and Wnt10b using BLAST and site‐directed mutagenesis of PCR, the pWnt10b‐mutant (Mut) vector was constructed. With renilla luciferase expression vector pRL‐TK (Takara Biotechnology Ltd., Dalian, China) serving as the internal control, the MEG3 vector or negative control (NC) was cotransfected with luciferase reporter vector respectively to HEK‐293T cells for detection of double luciferase activity using the Dual‐Luciferase Reporter Assay System (Promega, Madison, WI, USA). The experiment was repeated three times.

### 2.4 Subcellular location

The osteoblast strain MC3T3‐E1 was purchased from American Type Culture Collection (ATCC, Manassas, VA, USA). Fluorescence in situ hybridization (FISH) was employed in order to determine the subcellular location of lncRNA MEG3 in MC3T3‐E1 according to the instructions of the FISH Tag™ RNA Green Kit (RiboBio Co., Ltd., Guangzhou, Guangdong China) as follows: the cover glass was placed in a 6‐well culture plate and MC3T3‐E1 was inoculated in each well of the plate. After 1 day of culture, when cell confluence reached 80%, the cells were rinsed with phosphate buffer saline (PBS) and fixed with 1 mL 4% polyformaldehyde at room temperature. After treatment with protease K (2 μg/mL), glycine and phthalide reagent, the cells were incubated with 250 μL of pre‐hybridization solution at 42°C for 1 hour. With the removal of the pre‐hybridization solution, the cells were hybridized with 250 μL if hybridization solution containing probe (300 ng/mL) overnight at 42°C and rinsed with Phosphate‐Buffered Saline/Tween (PBST) three times. Next, the cell nucleus was stained using 6‐diamidino‐2‐phenylindole (DAPI) diluted by PBST. Inoculated in the 24‐well plate, the cells were stained for 5 minutes, rinsed with PBST three times, 3 minutes for each, mounted with anti‐fluorescence quenching agent and observed under a fluorescence microscope (Olympus, Tokyo, Japan) with five different visual fields selected and photographed.

### 2.5 Experimental animals

A total of 14 type II procollagen (Col2al)‐ICAT transgenic mice (aged about 8 weeks) with the expression of ICAT [inhibitor of β‐catenin and T cell factor (TCF)] driven by the Col2al promoter and 21 WT C57BL/6J mice (about 8 weeks old) were included in the current study. Col2al‐ICAT transgenic mice were constructed using gene targeting, provided by Professor Chen Di from the University of Rochester. C57BL/6J mice were obtained from the Laboratory Animal Center of Shanghai Institutes for Biological Sciences (Shanghai, China). All mice included in the current study were raised in cages with relatively stable temperature and humidity and with free access to water and food. Individual cages were manually changed twice a week.

### 2.6 Model establishment and grouping

The TF was performed at the middle of the right tibia of Col2al‐ICAT transgenic mice and C57BL/6J mice. After injections with 2% nembutal (WS20051129, Sinopharm Chemical Reagent Co., Ltd, Shanghai, China) for anesthetization, the mouse right tibia was exposed. After transection of the middle of the tibia, a bone nail (0.45 mm in diameter) was used to fix it and then the wound was closed. The following day, X‐ray examination was carried out to verify successful model establishment. A total of 21 C57BL/6J mice were assigned into the TF group [after establishment of TF, C57BL/6J mice were injected with 10 μL of phosphate buffer saline (PBS) at the site of the fracture], the siRNA group [after establishment of TF, C57BL/6 J mice were injected with 10 μL of MEG3 siRNA (4 mg/kg, MEG3 siRNA was synthesized by Sangon Biotech (Shanghai, China) at the site of the fracture] and the scramble group (after establishment of TF, C57BL/6J mice were injected with 10 μL of scramble siRNA at the site of the fracture). A total of 14 Col2al‐ICAT transgenic mice were assigned into the Col2a1‐ICAT group (after establishment of TF, Col2al‐ICAT transgenic mice were injected with 10 μL of PBS at the site of the fracture) and the Col2a1‐ICAT +siRNA group (after establishment of TF, Col2al‐ICAT transgenic mice were injected with 10 μL of MEG3 siRNA [4 mg/kg] at the site of the fracture).

### 2.7 X‐ray examination

X‐ray films, which showed a fracture of the right lateral tibia of mice, were imaged using a digital X‐ray imaging system (30 kV, 8 mAs) on the 21st day. In addition, the fracture healing and callus growth in mice were observed.

### 2.8 Safranin‐O/fast green staining

After X‐ray imaging was performed, the mice were killed and the tibia after fracture was extracted, fixed in 4% paraformldehyde, decalcified with 15% ethylenediaminetetraacetate (EDTA) (CAS: 60‐00‐4, Lishui Brandt Chemical Co., Ltd, Lishui, China), dehydrated with gradient alcohol and embedded in paraffin. The samples were sliced into 0.5 mm sections and stained with Safranin‐O/fast green. Next, the samples were dewaxed (paraffin sections were baked for half an hour at 90°C, dewaxed with xylene for 5‐10 minutes, washed with gradient alcohol with concentration of 100%, 95%, 90%, 80% and 70% for 3‐5 minutes and then washed with distilled water for 3 minutes), hydrated, stained with haematoxylin for 10 minutes, rinsed with water for 10 minutes, stained with fast green FCF (YM‐S1726, Shanghai Yuanmu Biological Technology Co., Ltd., Shanghai, China) for 5 minutes, immersed in 1% acetic acid for 10‐15 seconds, stained with 0.1% Safranin‐O (CAS:477‐73‐6, Shenzhen Kang Chu Yuan Co., Ltd. Shenzhen, China) for 5 minutes, dehydrated with 95% alcohol, 100% alcohol and xylene in order (twice, 2 minutes). After being mounted with resin, the samples were dried in a ventilated drying oven and stored at room temperature. The samples were observed using Nikon Eclipse 90i (Nikon Instruments Inc, Melville, NY).

### 2.9 Haematoxylin‐eosin (HE) staining

After X‐ray imaging was performed, the mice were killed and the tibia after fracture was extracted and fixed in 10% neutral buffered formalin (NBF) for 3 days. After that, the samples were rinsed with deionized water 3‐5 times and decalcified with 14% ethylene diamine tetraacetic acid (EDTA) solution for 14 days, with solution changed every other day. After decalcification, the samples were stained with HE as follows: the sections were dewaxed using xylene, dehydrated with ethanol of 100%, 95%, 85% and 70%, treated with 1% hydrochloric acid/ethanol, 1% haematoxylin, 0.5% ammonia and 95% ethanol. Next, the samples were dehydrated using ethanol of 70%, 90% and 100%. After vitrification with dimethylbenzene, the samples were mounted with neutral resin and then observed under an optical microscope (DSX100, Olympus, Beijing, China).

### 2.10 Immunohistochemistry

The paraffin‐embedded sections were dewaxed, added with bovine serum albumin (BSA) sealing fluid, primary antibodies rabbit anti‐collagen II (1:100, ab34712) and osteocalcin (1:100, ab76690) and secondary antibody biotin‐labelled goat anti‐rabbit IgG (1:1000, ab6721). All the antibodies were purchased from Abcam Inc (Cambridge, UK). Next, the sections were added with streptomycin avidin‐peroxidase solution, developed with diaminobenzidine (DAB) avoiding exposure to light and counterstained with haematoxylin. Lastly, the sections were routinely dehydrated using alcohol, cleared, mounted using neutral balsam and observed under a microscope.

### 2.11 Histomorphometrical analysis

Callus morphology, location and properties were observed and photographed under a microscope (100×). Magnification = objective magnification × eyepiece magnification. Histomorphometrical analysis was conducted using the Osteo‐measure software. Subsequently, the percentage of cartilage and bony callus area was recorded (Cg.Ar/Ps.Cl.Ar%, Md.Ar/Ps.Cl.Ar%).^23^, ^24^

### 2.12 Biomechanical analysis

The samples of fracture area at each time interval were gauze‐wrapped with physiological saline and then preserved at −80ºC. After thawing, the bone nail was removed and the distal and proximal end of the tibia was trimmed. It was ensured that the samples were kept moist during preparation and testing. Next, the samples were placed in the Instron material testing machine (INSTRON 5966, Instron Corporation, Boston, USA). The maximum flexural load (N) was measured using the asymmetric four‐point bending test^9^, ^25^ with span length set to 1 cm and loading speed set to 1 mm/min.

### 2.13 Nucleoplasm separation

Cytoplasm and nucleus was separated using kit (Thermo Scientific, MA, USA). Cells were centrifuged, lysed with 100‐500 μL pre‐cooled cell disruption buffer, ice‐bathed for 5‐10 minutes, centrifuged at 500 *g* for 1‐5 minutes at 4°C, with cytoplasmic layer extracted to Rnase‐free tube. The nucleus was washed with pre‐cooled cell disruption buffer and centrifuged at 500 *g* for 1‐5 minutes at 4°C with the supernatant discarded. After separation, the nucleus was used for RNA extraction.

### 2.14 Reverse transcription quantitative polymerase chain reaction (RT‐qPCR)

On the 21st day after fracture, the callus was incised 0.3 cm above and below the right TF and then ground to powder after the addition of liquid nitrogen. Total RNA was extracted from the callus tissues using the Trizol one‐step method following the instructions of Trizol (Invitrogen Inc, Carlsbad, CA, USA). Absorbance at wavelengths of 260 and 280 nm was measured using ND‐1000 UV/visible spectrophotometer (Thermo Scientific, Massachusetts, USA). Total RNA was quantified and accordingly the RNA concentration was adjusted. The extracted RNA was reverse transcribed into cDNA using the two‐step method following the manufacturer's instruction and the cDNA was preserved at −80ºC. RT‐qPCR was performed with the TaqMan probe method in accordance with the instructions of the kit (MBI Company, Lithuania). Primer sequences are shown in Table 1. The reaction conditions were as follows: pre‐denaturation for 30 seconds at 95°C, 40 cycles of denaturation for 10 seconds at 95°C, annealing for 20 seconds at 60°C and extension for 10 seconds at 79°C. The reaction system was as follows: 12.5 μL Premix Ex Taq or SYBR Green Mix, 1 μL Forward Primer (80 pmol/μL), 1 μL Reverse Primer (80 pmol/μL), 1‐4 μL DNA template and 25 μL ddH_2_O. Real time PCR instrument (Bio‐Rad iQ5, Bio‐Rad Company, San Francisco, USA) was used for detection. β‐actin was regarded as the internal reference and the fold changes between the experiment group and the control group were calculated by means of the relative quantification 2^–△△Ct ^method.^26^ The experiment was independently repeated three times to obtain the mean value.

**Table 1:** Primer sequences for RT‐qPCR

Gene	Primer sequence (5′‐3')
Col10a1	Reverse: GCAGCATTACGACCCAAGAT
	Forward: CATGATTGCACTCCCTGAAG
Runx2	Reverse: TTTAGGGCGCATTCCTCATC
	Forward: TGTCCTTGTGGATTAAAAGGACTTG
Osterix	Reverse: TCTCAAGCACCAATGGACTCCT
	Forward: GGGTAGTCATTTGCATAGCCAGA
Osteocalcin	Reverse: GACAAAGCCTTCATGTCCAAGC
	Forward: AAAGCCGAGCTGCCAGAGTTTG
MEG3	Reverse: GGGAGCAGCTATGGATCACC
	Forward: ATAGCGCCCCCTATTCATGC
β‐actin	Reverse: CGAGCTCTGAGCACTGGAGA
	Forward: TGGCGTGTAAAGTCACCACC

RT‐qPCR, reverse transcription quantitative polymerase chain reaction; Runx2, Runt‐related transcription factor 2; MEG3, maternally expressed gene 3.

### 2.15 Western blot analysis

On the 21st day after fracture, 3 mL of lysate [7 mol/L urea, 2 mol/L sulphourea, 5 mL/L IPG buffer (pH 3 ~ 10), 65 mmol/L dithiothreitol (DTT), 40 g/L 3‐[(3‐cholidopropyl) dimethylammonio]‐1‐propansulfanate (CHAPS), 5 mg/L protease inhibitor and 10 mL/L trypsin inhibitor] were added and ultrasonicated on ice to extract proteins. Next, the protein concentration was measured using the bicinchoninic acid (BCA) method, followed by electrophoresis with polyacrylamide gel (5% spacer gel and 12% separation gel) and transferred to the membrane. Subsequently, the membrane was blocked for 1 hour at room temperature with tris‐buffered saline tween (TBST) buffer containing 5% BSA on a bleaching shaking table. The primary antibodies were prepared with 5% BSA. Next, the membrane was incubated at 4ºC overnight with following primary antibodies: rabbit Co110a1 (1:200, ab58632), rabbit anti‐Runx2 (1:1000, ab23981), rabbit anti‐Osterix (1:1000, ab94744), rabbit anti‐Osteocalcin (1:500, ab93876), rabbit anti‐wnt10b (1:500, ab70816), rabbit anti‐p‐GSK‐3β (1:500, ab75745), rabbit anti‐GSK‐3β (1:500, ab131356), rabbit anti‐β‐catenin (1:5000, ab184947), rabbit anti‐p‐β‐catenin (1:500. ab27798) and rabbit anti‐β‐actin (1:1000, ab5694). Next, the membrane was added with the secondary antibody goat anti‐rabbit (ab6721) and incubated at 4ºC for 4‐6 hours. All the antibodies mentioned above were purchased from Abcam Inc (MA, USA). Then, the membrane was washed with TBST (3 × 15 minutes). The chemiluminescence reagent A and B (Shanghai Yanhui Biotechnology Co., Ltd, Shanghai, China) were mixed at the ratio of 1:1 and then added into the membrane for developing. Finally, the densitometric analysis was performed in all immunoblot bands. The experiment was independently repeated three times to obtain the mean value.

### 2.16 Statistical analysis

Statistical analyses were performed with the SPSS 21.0 statistical software (IBM, Armonk, NY, USA). Measurement data were expressed by mean ± standard deviation. Data among multiple groups were compared with two‐way analysis of variance and comparisons among the two groups were performed with the *t* test. *P < *0.05 indicated a statistically significant difference.

## 3 RESULTS

### 3.1 MEG3 is up‐regulated in non‐union fracture bone

**Figure 1:**
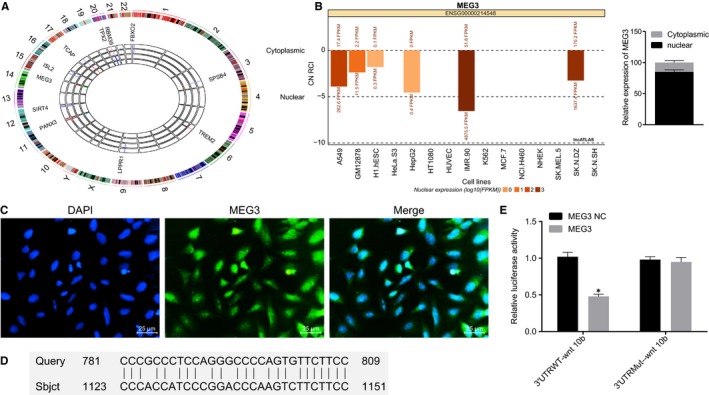
MEG3 is up‐regulated in non‐union bone fracture. A, RCrico plot of non‐union bone fracture‐related chip GSE494 (from GEO database in NCBI), in which number 1‐22, X and Y referred to different chromosomes. The outmost circle represents 22 chromosomes, X and Y. The sideward four circles represent four samples. From outside to inside, the four circles refer to healing bone samples, healing bone samples, non‐union fracture bone and non‐union fracture bone, independently. B, location of MEG3 predicted in the LncATLAS website and quantitative analysis of MEG3 in the nucleoplasm. C, location analysis of MEG3 in cells detected using FISH. The pre‐osteoblast cell strain MC3T3‐E1, a classical cell model to study osteoblasts, was used for subcellular location. D, complementary pairing sequence between promoter region of Wnt10b and MEG3. E, the luciferase activity of 3ʹUTRWT‐wnt10b and 3ʹUTRMut‐wnt 10b. MEG3, maternally expressed gene 3; FISH, Fluorescence In Situ Hybridization; CN RCI, cytoplasmic nuclear relative concentration index; GEO, Gene Expression Omnibus; NCBI, National Center of Biotechnology Information

GSE494 chip data were analysed and the results indicated that MEG3 was up‐regulated in non‐union fracture bone samples when compared to that in healing fracture samples. In addition, MEG3 was found to be localized on Chromosome 15. Detailed RCircos plot is shown in Figure 1A. MEG3 gene showed mutation in Chromosome 15 and lncATLAS website displayed that MEG3 was located in the nucleus and the quantitative analysis for extracted RNA after nucleoplasm separation showed that MEG3 mainly expressed in nucleus (Figure 1B). Results of FISH revealed that fluorescence was primarily observed in the cell nuclei (Figure 1C). The RAID v2.0 website revealed that MEG3 could bind to methyltransferase enhancer of the zeste homolog 2 (EZH2).^27^ A previous study indicated that EZH2 could inhibit the expression of Wnt10b^28^ and results of BLAST showed that there was complementary pairing sequence between the promoter region of Wnt10b and MEG3 (Figure 1D). The results of dual‐luciferase reporter gene assay indicated that MEG3 could bind to the promoter region of Wnt10b (Figure 1E). These findings show that MEG3 is overexpressed in non‐union bone fracture and is very likely to participate in the pathological process of non‐union bone fracture.

### 3.2 Silencing MEG3 promotes fracture healing and callus growth

**Figure 2:**
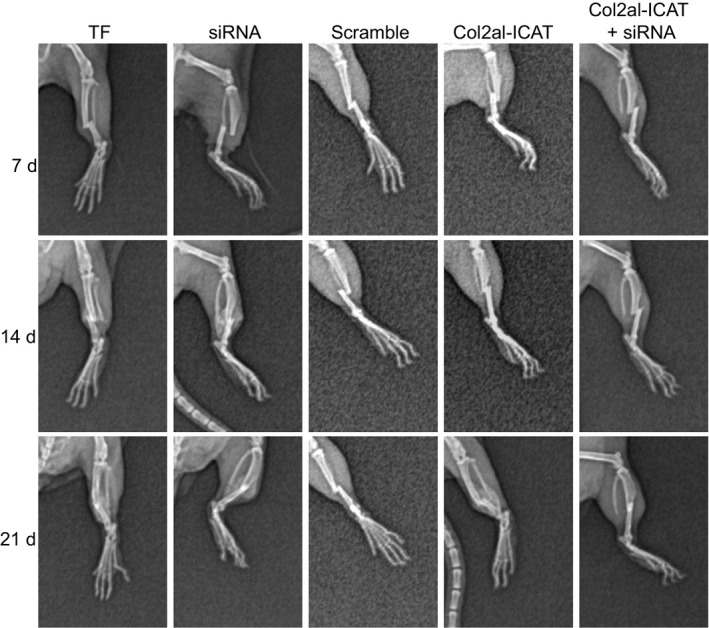
X‐ray imaging shows that silencing MEG3 promotes fracture healing. siRNA, small interfering RNA; Col2a1‐ICAT, type II procollagen‐inhibitor of β‐Catenin and T Cell Factor; MEG3, maternally expressed gene 3

X‐ray analyses were performed in order to verify the successful establishment of TF models. It was observed that the fracture lines of mice in each group were clearly visible on the 7th day after fracture, whereas soft callus appeared in mice in the siRNA group. On the 14th day after fracture, mice in the TF, scramble and Col2a1‐ICAT+siRNA groups showed blurred fracture lines, mice in the siRNA group showed improved callus formation improved and healing fracture and mice in the Col2a1‐ICAT group showed slightly blurred fracture lines and callus. On the 21st day after fracture, there appeared to be blurred fracture lines and bridge callus of mice in the TF, scramble and Col2a1‐ICAT +siRNA groups, complete callus formation and basic fracture healing in the siRNA group and blurred fracture line and callus in the Col2a1‐ICAT group without bridge callus (Figure 2).

### 3.3 Silencing MEG3 accelerates reconstruction of TF in mice

**Figure 3:**
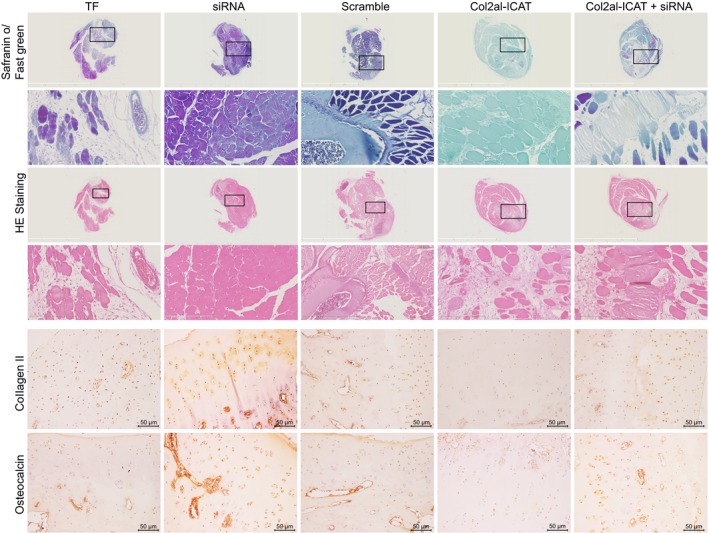
Safranin‐O/Fast green (original magnification of 200×), HE staining (original magnification of 400×) and immunohistochemistry (original magnification of 200×) show that silencing MEG3 promotes the callus growth at 21st day after tibia fraction. MEG3, maternally expressed gene 3; HE, haematoxylin‐eosin

Safranin‐O/Fast green and HE staining were adopted in order to observe the fracture healing and callus growth of mice with different treatment regimens (Figure 3). Safranin‐O/Fast green showed that on the 21st day after fracture, the callus was in the reconstruction stage and multiple relic woven bones in ossis existed of mice in the TF, scramble and Col2a1‐ICAT +siRNA groups. The callus was observed to be in the reconstruction stage and the ossis maintained repatency with few relic woven bones in the siRNA group. Mice in the Col2a1‐ICAT group presented with spongiose trabecular bone and a great number of callus cartilage and osseous callus around the site of fracture.

Similarly, HE staining showed that on the 21st day after fracture, the callus was in the reconstruction stage and multiple relic woven bones in ossis existed in the TF, scramble and Col2a1‐ICAT +siRNA groups. The callus was observed to be in the reconstruction stage and the ossis maintained repatency with more compact and regular shapes and with more relic woven bones in the siRNA group and mice in the Col2a1‐ICAT group presented with spongiose trabecular bone and a great number of callus cartilage and osseous callus around the site of fracture.

The results of immunohistochemistry on the 21st day after fracture showed that compared with the TF group, the positive expression of collagen II and osteocalcin was significantly increased in the siRNA group whereas it was decreased in the Col2a1‐ICAT group (all *P* < 0.05) and no significant difference was found in the scramble and Col2a1‐ICAT +siRNA groups (*P* > 0.05).

The aforementioned results verify that siRNA of MEG3 could accelerate reconstruction of TF in mice.

### 3.4 Silencing MEG3 contributes to faster fracture healing and functional recovery

**Figure 4:**
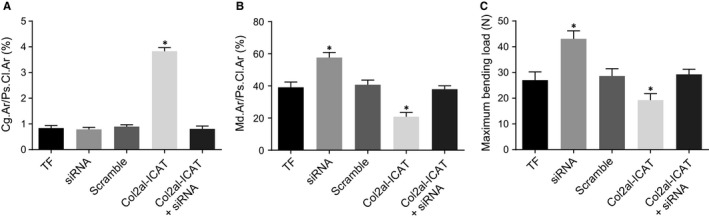
Histomorphometrical analysis and biomechanical analysis shows that silencing MEG3 contributes to faster fracture healing and functional recovery. A, Cg.Ar/Ps.Cl.Ar% among the TF, siRNA, scramble, Col2a1‐ICAT and Col2a1‐ICAT +siRNA groups. B, Md.Ar/Ps.Cl.Ar% among the TF, siRNA, scramble, Col2a1‐ICAT and Col2a1‐ICAT +siRNA groups. C, comparison of maximum flexural load of mice among the TF, siRNA, scramble, Col2a1‐ICAT and Col2a1‐ICAT +siRNA groups. MEG3, maternally expressed gene 3; TF, tibia fracture; siRNA, small interfering RNA; Col2a1‐ICAT, type II procollagen‐inhibitor of β‐Catenin and T Cell Factor; siRNA, small interfering RNA; Col2a1‐ICAT, type II procollagen‐inhibitor of β‐Catenin and T Cell Factor; Cg.Ar/Ps.Cl.Ar% indicates percentage of soft callus area; Md.Ar/Ps.Cl.Ar% indicates the percentage of osseous callus area; *P < 0.05 vs the TF group; mean ± standard deviation; *t* test is performed for two‐group comparisons; the two‐way analysis is performed for multiple‐group comparison

Histomorphometrical analysis was performed in order to study the percentage of soft callus area in TF mice after different treatment and furthermore, biomechanical analysis was carried out for analysis of functional recovery in TF mice (Figure 4A‐C). On the 21st day after fracture, no significant differences in percentage of soft callus area were observed between the TF group and the siRNA group (*P* > 0.05) but an increased percentage of osseous callus area was found in the siRNA group (all *P* < 0.05). There were no significant differences among the TF, scramble and Col2a1‐ICAT +siRNA groups (all *P* > 0.05). The percentage of soft callus area increased and the percentage of osseous callus area decreased in the Col2a1‐ICAT group (all *P* < 0.05) compared with the TF group. On the 21st day after fracture, compared with the TF group, maximum flexural load was found to be increased in the siRNA group (*P* < 0.05). No significant difference was found among the TF, scramble and Col2a1‐ICAT +siRNA groups (*P* > 0.05), whereas a decreased maximum flexural load existed in the Col2a1‐ICAT group (*P* < 0.05). All the above results imply that siRNA of MEG3 may accelerate fracture healing and advance functional recovery in TF mice.

### 3.5 Silencing MEG3 increases expression of Col2a1, Runx2, Osterix and Osteocalcin

**Figure 5:**
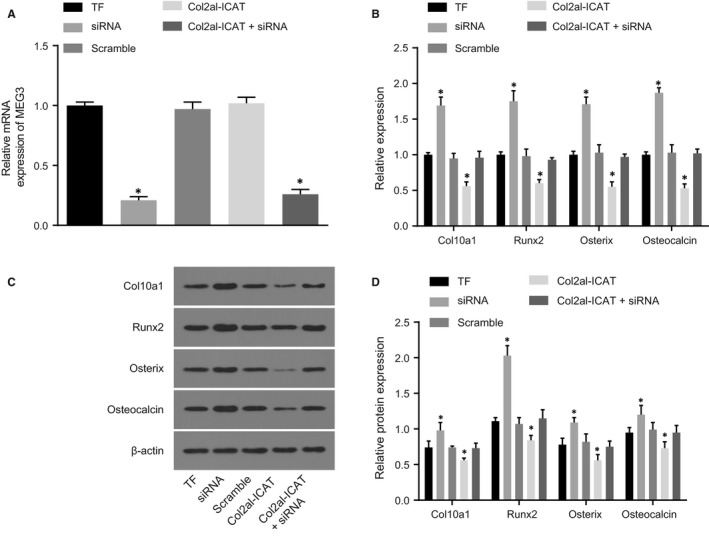
Silencing MEG3 increases expression of Col10a1, Runx2, Osterix and Osteocalcin. A, MEG3 expression in callus tissues in each group. B, mRNA expression of Col10a1, Runx2, Osterix and Osteocalcin in each group. C, protein bands of Col10a1, Runx2, Osterix, Osteocalcin and β‐actin in each group. D, protein levels of Col10a1, Runx2, Osterix, Osteocalcin. TF, tibia fracture; Runx2: Runt‐related transcription factor 2; MEG3, maternally expressed gene 3; siRNA, small interfering RNA; Col2a1‐ICAT, type II procollagen‐inhibitor of β‐Catenin and T Cell Factor; RT‐qPCR, reverse transcription quantitative polymerase chain reaction; *P < 0.05 vs the TF group; mean ± standard deviation; *t *test is performed for two‐group comparisons; the two‐way analysis is performed for multiple‐group comparison

RT‐qPCR was employed in order to measure the expression of MEG3 in callus tissues and the results (Figure 5A) showed that in comparison with the TF group, MEG3 was decreased in the siRNA and Col2a1‐ICAT +siRNA groups but showed no obvious difference in the scramble and Col2a1‐ICAT groups. In addition, mRNA and protein expression of cartilage and bone‐related marker genes in callus was detected using RT‐qPCR and Western blot analysis (Figure 5B‐D). Compared with the TF group, the expression of Col10a1, Runx2, Osterix and Osteocalcin increased in the siRNA group but decreased in the Col2a1‐ICAT group (all *P* < 0.05). All findings demonstrate that silencing of MEG3 promotes fracture healing.

### 3.6 Silencing MEG3 activates the Wnt/β‐catenin signalling pathway

**Figure 6:**
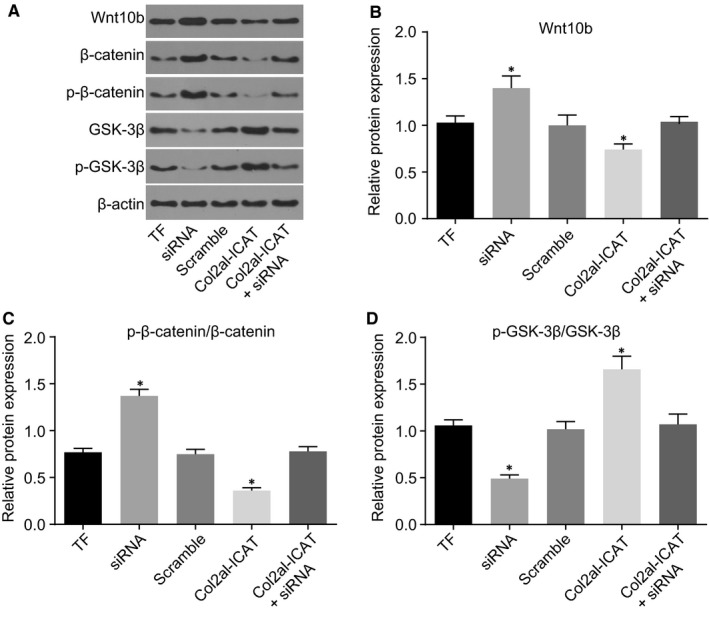
Silencing MEG3 activates the Wnt/β‐catenin signalling pathway. A, the protein bands of Wnt10b, p‐β‐catenin, β‐catenin, p‐GSK‐3β, GSK‐3β and β‐actin in each group. B‐D, the protein expression of Wnt10b, β‐catenin and GSK‐3β in each group. MEG3, maternally expressed gene 3; siRNA, small interfering RNA; GSK‐3β, glycogen synthase kinase‐3β; MEG3, maternally expressed gene 3; TF, tibia fracture; *P < 0.05 vs the TF group; mean ± standard deviation; *t *test is performed for two‐group comparisons; the two‐way analysis is performed for multiple‐group comparison

Finally, Western blot analysis was performed in order to investigate the effects of siRNA of MEG3 on the Wnt/β‐catenin signalling pathway (Figure 6A‐D). No significant difference in protein levels of Wnt10b, p‐β‐catenin/β‐catenin and p‐GSK‐3β/GSK‐3β was detected between the TF and scramble groups (all *P* > 0.05). Compared with the TF group, the siRNA group showed increased protein levels of Wnt10b and p‐β‐catenin/β‐catenin but decreased p‐GSK‐3β/GSK‐3β (*P* < 0.05) protein levels and the Col2a1‐ICAT group exhibited decreased protein levels of Wnt10b and p‐β‐catenin/β‐catenin (*P* < 0.05) but increased GSK‐3β protein levels (*P < *0.05). Compared with the TF group, there were no significant differences in the protein levels of Wnt10b, p‐β‐catenin/β‐catenin and p‐GSK‐3β/GSK‐3β in the scramble and Col2a1‐ICAT +siRNA groups and no significant difference in the MEG3 protein levels in the scramble group (all *P* > 0.05). The results revealed that siRNA of MEG3 could activate the Wnt/β‐catenin signalling pathway.

## 4 DISCUSSION

Currently, the most accepted theory of fracture healing is blood in fracture site becoming healed bone.^29^ There are several ways in which bones could recover, whereas the distinctive feature of bony repair lies in its occurrence without the development of a fibrous scar, indicating that the process is also a form of tissue regeneration.^30^ However, the proportion of delay and non‐union is reported to be the highest in fracture cases, frequently associated with severe trauma or loss of surrounding muscle tissue.^31^ Therefore, in the current study, through the combination of in vivo experimentation and cell experimentation, we found that epigenetic silencing of MEG3 could promote fracture healing by activating the Wnt/β‐catenin signalling pathway.

Firstly, the current study reported the finding that MEG3 is up‐regulated in non‐union fracture bone. Interestingly, it has been previously reported that lncRNA HOXA11‐AS is involved in fracture healing due to its effects on cell proliferation and apoptosis.^32^ A previous study proved that MEG3 could inhibit proliferation and promote apoptosis of tumour cells.^33^ Furthermore, blurring of fracture margins and reactive sclerosis are known to be the earliest signs of healing in both modalities.^34^ The fracture callus is radially composed of oriented spokes of woven bone in a cartilage matrix and the original cortical bone before the fracture largely erodes.^35^ Moreover, the current study revealed that silencing MEG3 promotes ossis repatency and callus reconstruction, which suggests that silencing MEG3 promotes fracture healing.

In addition, our results showed that silencing MEG3 increases the expression levels of Col10a1, Runx2, Osterix and Osteocalcin. COL2A1 was found to be associated with cartilage formation, in accordance with our results that on the 7th day after fracture, multiple cartilage cells and large callus area were observed in the siRNA group. Runx2 and osterix are osteoblast‐related genes and phosphatase alkaline and osteocalcin belong to the family of osteoblast marker proteins.^36^ It is known that COL10A1 is a form of short‐chain collagen of cartilage, synthesized by chondrocytes during the process of growing long bones.^37^ In addition, Runx2 and osterix are known to regulate the osteogenic differentiation of mesenchymal pluripotent cells and the expression of various bone marker genes.^38^ LncRNA KCNQ1OT1 has been proven to up‐regulate Runx2, Osterix and Osteocalcin, suggesting that KCNQ1OT1 serves as a potential positive mediator of osteoblastic differentiation.^19^ These previous studies support that Col10a1, Runx2, Osterix and Osteocalcin expressions are associated with bone formation and elevated expression levels of Col10a1, Runx2, Osterix and Osteocalcin indicate towards fracture healing. Therefore, silencing MEG3 increases the expression levels of Col10a1, Runx2, Osterix and Osteocalcin, suggesting that silencing MEG3 promotes fracture healing. Furthermore, Col10a1, Runx2, Osterix and Osteocalcin are all transcriptionally regulated by β‐catenin. Similarly, Ioanna et al verified that COL10A1 expression was regulated via activation of the Wnt/β‐catenin signalling pathway,^39^ which was also found to participate in osteogenic transdifferentiation and arterial medial calcificatio by directly targeting Runx2.^40^ In addition, the inactivated Wnt signalling pathway could decrease the expression of each osteogenic transcription factor, including osterix and osteocalcin,^41^ which is in line with the results of the current study.

Lastly, the obtained data implied that silencing MEG3 increases the expression levels of Wnt10b and β‐catenin but decreases GSK‐3β expression level, suggesting that MEG3 knockdown can activate the Wnt/β‐catenin signalling pathway. LncRNAs could perform their function via molecular and biochemical mechanisms including cis‐ and trans‐regulation of gene expression, post‐transcriptional control and epigenetic modulation in the nucleus in the cytoplasm.^14^ The Wnt pathway can be stimulated via inhibitors of glycogen synthase kinase‐3β (GSK‐3β) GSK‐3β leading to increased β‐catenin, which plays vital roles in chondrocyte development and maturation.^42^ Moreover, it is reported that epigenetic silencing of lncRNA MEG3 could promote fracture healing by inhibiting β catenin/survivin by activating p53.^33^ In addition, a previous study demonstrated that Wnt/β‐catenin signalling could mediate mutual inhibition among the genetic programs, leading to differentiation of chondrocytes or osteoblasts in bone cartilage.^43^ During skeletal development of embryo, suppression of β‐catenin signalling prevents osteoblastic differentiation and then induces development of pluripotent mesenchymal cells to the chondroblastic phenotype, verifying that the Wnt/β‐catenin pathway could be activated during fracture healing in both animals and humans.^20^ These evidences support that silencing MEG3 promotes fracture healing via activation of the Wnt/β‐catenin signalling pathway.

**Figure 7:**
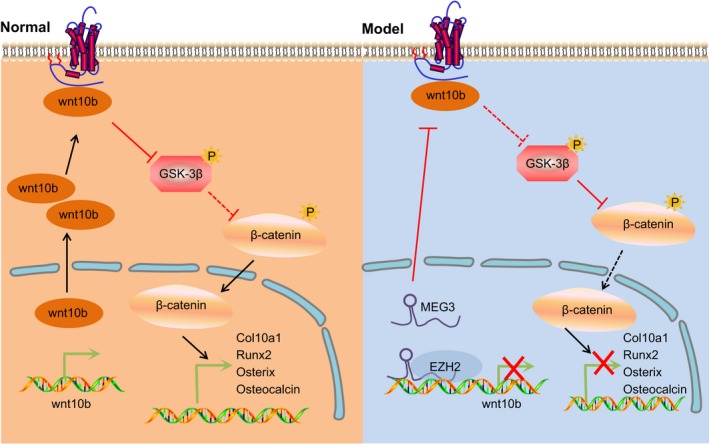
The molecular mechanism involved lncRNA MEG3 affecting the fraction healing of mice after tibia fracture. Silenced MEG3 increased the expression of Col10a1, Runx2, Osterix, Osteocalcin, Wnt10b and β‐catenin whereas it decreased the expression of GSK‐3β, thus improving fracture healing. Lnc, long non‐coding RNA; MEG3, maternally expressed gene 3; GSK‐3β, glycogen synthase kinase‐3β

In summary, the current study demonstrates that epigenetic silencing of MEG3 promotes fracture healing by activating the Wnt/β‐catenin signalling pathway (Figure 7). However, few limitations in the present investigation still remain such as the small sample size and the lack of understanding of MEG3 molecular mechanism during the process of facture healing in rats . Therefore, continuous improvement and relevant research are warranted in the future.

## CONFLICT OF INTEREST

None.
